# Response and resilience of soil microbial communities inhabiting in edible oil stress/contamination from industrial estates

**DOI:** 10.1186/s12866-016-0669-8

**Published:** 2016-03-22

**Authors:** Vrutika Patel, Anukriti Sharma, Rup Lal, Naif Abdullah Al-Dhabi, Datta Madamwar

**Affiliations:** Post Graduate Department of Biosciences, Centre of Advanced Study in Bioresource Technology, Sardar Patel University, Satellite Campus, Vadtal Road, Bakrol, 388 315 Gujarat India; Department of Zoology, University of Delhi, Delhi, India; Department of Botany and Microbiology, Addiriya Chair for Environmental Studies, College of Science, King Saud University, P.O. Box # 2455, Riyadh, 11451 Saudi Arabia

**Keywords:** Oil perturbation, β-proteobacteria, 16S rRNA gene, Bacterial community structure, Fatty acid biosynthesis, Enzymes

## Abstract

**Background:**

Gauging the microbial community structures and functions become imperative to understand the ecological processes. To understand the impact of long-term oil contamination on microbial community structure soil samples were taken from oil fields located in different industrial regions across Kadi, near Ahmedabad, India. Soil collected was hence used for metagenomic DNA extraction to study the capabilities of intrinsic microbial community in tolerating the oil perturbation.

**Results:**

Taxonomic profiling was carried out by two different complementary approaches i.e. 16S rDNA and lowest common ancestor. The community profiling revealed the enrichment of phylum “*Proteobacteria*” and genus “*Chromobacterium*,” respectively for polluted soil sample. Our results indicated that soil microbial diversity (Shannon diversity index) decreased significantly with contamination. Further, assignment of obtained metagenome reads to Clusters of Orthologous Groups (COG) of protein and Kyoto Encyclopedia of Genes and Genomes (KEGG) hits revealed metabolic potential of indigenous microbial community. Enzymes were mapped on fatty acid biosynthesis pathway to elucidate their roles in possible catalytic reactions.

**Conclusion:**

To the best of our knowledge this is first study for influence of edible oil on soil microbial communities via shotgun sequencing. The results indicated that long-term oil contamination significantly affects soil microbial community structure by acting as an environmental filter to decrease the regional differences distinguishing soil microbial communities.

**Electronic supplementary material:**

The online version of this article (doi:10.1186/s12866-016-0669-8) contains supplementary material, which is available to authorized users.

## Background

Oil spills have been pivotal in delineating microbial diversity at the affected site in contrast to the pristine soil. Thus, it becomes important to understand how indigenous microbial communities respond to the stress in order to understand their role in degradation process. Implementation of efficacious bioremediation strategies relies on innate microbial community dynamics, structure, and function [1]. Depending on biotic and abiotic factors, microorganisms adapt to the environment and accordingly environmental conditions select for microorganisms featuring specific capabilities. Other environmental variables also influence microbial distribution, such as regional climate [2, 3] soil type and characteristics [4] and vegetation [5].

Interestingly, microbial community interacts with each other to adapt under extreme environmental changes via modulating genome architecture [6, 7]. The vast majority of these organisms have been characterized through culture-independent molecular surveys using conserved marker genes like the small subunit ribosomal RNA or more recently the shotgun sequencing [8, 9]. The ongoing development of next generation sequencing (NGS) methods can now be combined with advanced bioinformatics method to replace more traditional approach of metagenomic library screening [10–12]. Consequently, more and more complete microbial genomes as well as environmental metagenomes are being sequenced to gain insights into functional aspects besides species composition [13].

Till date microbial community studies in oil-polluted sites have been carried out either for crude oil contamination (marine and coastal sites) [14–16] or petroleum oil contamination [17, 18] or desert soils [19, 20] but limited efforts have been made in the direction of studying role of bacterial community at sites tainted with edible oil. Consequently, the intrinsic microbial community has to be active degrader organic pollutants (fats and lipids). The process of biodegradation mainly depends on microorganisms which enzymatically attack the pollutants and convert them into innocuous products [21]. Major group of extracellular hydrolytic enzymes disrupt chemical bonds in the toxic molecules and results in the reduction of their toxicity. This mechanism is effective for biodegradation of oil spill as well as organophosphate and carbamate insecticides. Bacteria such as *Bacillus sp.*, *Pseudomonas sp.*, *Chromobacterium vinosum* and the fungi *Aspergillus niger* and *Humicola lanuginose, Rhizopus delemar* and *Candida rugosa* secrete hydrolytic enzyme i.e. lipase that hydrolyse triacylglycerides to fatty acids and glycerol [22] and catalyze the degradation of lipids. Recent reports have shown that lipase activity is closely related with organic pollutants present in the soil and for the drastic reduction of total hydrocarbon from contaminated soil. Hence, research undertaken in this area is likely to progress the knowledge in the bioremediation of oils spill [23].

The study aims at the metagenomic-analysis of the microbial community inhabiting long-term edible oil contaminated site for both taxonomic profile and catabolic gene potential. Taxonomic profiling will provide insights into the composition of the microbial community capable of tolerating and/or degrading fatty acid compounds. Functional characterization of metagenome sequence reads on the basis of Clusters of Orthologous Groups of proteins (COG) accessions and Kyoto Encyclopedia of Genes and Genomes (KEGG) database entries will lead to elucidation of the catabolic potential of the indigenous microbial community. This approach will facilitate identification of genes essential for key catalytic steps in biodegradation pathways with respect to edible oil. Concisely, the obtained data will improve our understanding for the dynamics of bacterial community inhabiting oil stress and will also assess the genomic potential of the indigenous microbial community of the contaminated soil habitat.

## Methods

### Survey of the sampling site and physicochemical analysis of soil samples

To study the shift in microbial community structure across the oil polluted sites we collected bulk soil samples from the depots of oil contamination located near industrial area of Kadi, Ahmedabad. Three different sampling sites (i.e. P1, P2, P3) of soil were selected that represents accumulated edible cotton seed oil contamination since 20 years, resulted from oil spillage in ginning mills (GPS location for polluted site 23 degrees 17′46.2624″N_72 degrees 20′37.2840″E). Another sampling site was located within the industrial estate area 500 meter without any contamination i.e. control soil sample (C1, C2, C3) and was considered as a reference to demonstrate changes in microbial community under oil stress (GPS location for control site 23 degrees 17′17.1780″N_72 degrees 21′36.6048″E). At each site sampling was performed in replicates and collected soil was archived at 4 °C until further use. Physicochemical analysis of soil samples such as soil moisture, soil texture, organic carbon content, soil carbon/nitrogen ratio (C:N) were determined. This soil sample is collected from the soil where the cluster of edible oil industries are available, does not involve any ethical issues. No prior permission was required as this land does not belong to any specific agency. However, field studies do not create any destruction to endangered or protected species.

### Community DNA extraction and sequencing

Metagenomic DNA from each soil samples i.e. polluted and control (P1, P2, P3, C1, C2 and C3) was extracted using protocol described by Zhou et al. [24]. In all 50 μL MilliQ water was used to dissolve DNA at the final step.

Soil sample (5 g) was pre-washed with double distilled water before DNA extraction according to the method of Zhou et al. [24]. Briefly, after adding 5 g glass beads (d = 3 mm) and 15 mL DNA extraction buffer (100 mM Tris, 100 mM EDTA, 1.5 M NaCl, 10 % Sucrose, 1 % CTAB, 100 mM sodium phosphate buffer pH = 8.0) to the pretreated soil, the sample was vortexed for 5 min followed by incubation for 30 min at 37 °C on environmental shaker. Subsequently, 2 ml SDS (20 %) was added and mixed with hand-shaking for 5 min. The sample was incubated at 60 °C for 30 min and inverted every 10 min. Further, 0.5 g of powdered activated charcoal (PAC) was added and incubated for 30 min more [25]. After centrifugation at 12000 rpm for 15 min at room temperature, DNA was extracted with an equal volume of phenol and chloroform-isoamyl alcohol (24:1, v/v), precipitated with isopropanol and washed with 70 % ethanol.

Total DNA concentration and quality was analyzed by NanoDrop spectrophotometer and electrophoresed on 0.8 % agarose gel, respectively. Equal concentration of environmental metagenomic DNA (obtained by Qubit reading) from each subsequent sites were mixed to form a composite genetic pool (i.e. P1 + P2 + P3 = P and C1 + C2 + C3 = C) representing total DNA composition for each site. Isolated DNA was sheared and sized to produce DNA library according to the manufacturer’s protocol from Ion Xpress™ Plus gDNA Fragment Library Preparation Kit. DNA Sequencing was performed on Ion Torrent PGM platform using sequencing chip 318 to generate short reads with an average insert size of 300 bp. All the outsourcing for DNA sequencing was done at Xcelris Lab Pvt Ltd, Ahmedabad, India.

### Assembly and taxonomic analysis for sequencing data

Sequences generated for polluted as well as control sample was assembled individually by MetaVelvet assembler (1.20.02) [26, 27] set at *k* = 31, −exp cov = auto, −cov_cutoff = auto and insertion length with standard deviation = 300 bp ± 20. Both raw reads and contigs were used for further analysis. The taxonomic positions of sequenced reads was analysed and studied using two complementary approaches: (1) LCA: classification based on lowest common ancestor using MEGAN [28] and (2) Ribosomal Database Project (RDP) classifier: classification based on 16S rRNA gene sequences. MEGAN platform uses the lowest common ancestor (LCA) algorithm to classify reads to certain taxa based on their blast hits [29, 30]. The LCA parameters were set as Min Score = 35.0, Top Percent = 50, and Min Support = 2. In addition, the 16S rRNA sequences were extracted from the results of BLASTN analysis against the nt/nr database [26] and submitted to the RDP classifier [29, 31] with E value < 1X10^−1^ and 80 % confidence level. The RDP classifier predicted the taxonomic origin of 16S rRNA up to the rank of genus. Moreover, in order to rectify diversity picture other reference databases such WebCARMA (based on Environmental gene tags i.e. EGTs) and non-redundant database M5NR were also used with standard parameters.

### Rarefaction analysis, Diversity indices and multivariate component analysis

Rarefaction curve was generated for all reads, except unassigned reads. Species richness was plotted according to the data obtained from RDP dataset [31], whereas, additional species likely to be discovered was addressed by plotting the discovery rate of dataset, which is obtained by repeatedly selecting random subsample of the dataset at 10, 20, upto- 90 % of the original size and then plotting the number of leaves predicted by LCA algorithm using MEGAN [28]. The diversity index i.e. Shannon’s evenness index for general diversity (at genus level) and Simpson’s dominance index on the basis of genus were calculated as described previously [25, 32, 33]. Multivariate principle component analysis (PCA), contour plot and correspondence analysis plot was plotted using data of phylum in PAST3 software [34].

### Mapping of metagenomic reads

Polluted metagenomic single reads were mapped on available microbial genomes by aligning to the sequenced genome(s). An E-value cut off of 1Xe^−3^ and log_2_ as abundance scale was set. The coverage of reference genome sequence by reads was visualized using the Circos [35, 36].

### Functional characterization and classification of genes

Functional characterization of reads was done on the basis of assembled data obtained from polluted sample. Gene calling was performed on the contigs using FragGeneScan [37] in order to predict operon reading frame (ORF). The ORFs were functionally annotated and assigned to the Clusters of Orthologous Groups of proteins (COG) [38] with an E value cut-off 10^−5^. The metabolism analysis was performed on KEGG Orthology (KO)-identifiers by using KAAS tool (KEGG Automatic Annotation Server) based on bi-directional best hit approach (60). Gene annotation was based on Enzyme Commission (EC)-numbers based on the Kyoto Encyclopedia of Genes and Genomes (KEGG) Orthology database [39].

### Screening of bacterial strains having oil degradation ability

Soil obtained from polluted sample was serially diluted and spread on Nutrient agar, Luria agar, Plate count agar and Tributyrin agar plates supplemented with tributyrin oil as a carbon source. Bacterial strains were selected on the bases of their ability to hydrolyze tributyrin oil by producing a clear zone around bacterial colonies [22, 40–42]. A total of 40 strains were isolated based on their distinct morphological characteristics of the colonies and hydrolysis of trybutyrin oil. All the isolated culture on plates was observed for 48 h and sub cultured once in every month. Further, all the bacterial strains were maintained at 4 °C in pure form for further use.

### Data availability

The sequence data for both soil samples i.e. polluted and control obtained from Ion Torrent PGM platform has been deposited at MGRAST server (version 3). MGRAST IDs for the datasets are 4508969.3 and 4516462.3 for polluted soil and control soil, respectively. MGRAST IDs for the contig obtained from both the samples are 4515485.3 and 4512472.3, respectively. The sequences obtained from the culturable diversity study have been submitted to GenBank, NCBI and their accession numbers are from KR140170 to KR140186 (polluted soil) and KR140187 to KR140201 (control soil).

## Results

Multiple studies have demonstrated the applications of high throughput sequencing in the study of microbial distributions and functional genes in different microbial communities. However, there are limited reports on bacterial community structural studies for edible oil contamination sites. Therefore, we took this opportunity to explore effect of oil on bacterial community shift in long term edible oil-contaminated sites from industrial area of Kadi, Ahmedabad. Innate microbial community inhabiting contaminated environment was analyzed in terms of its composition and diversity by high-throughput shotgun sequencing approach via ion-torrent PGM platform provided by Xcelris Lab Pvt Ltd, Ahmedabad, India.

### Physicochemical analysis of soils

The physicochemical analysis of soil samples from both (control and polluted) sites are tabulated in Table 1 showing significant difference (*P-value* <0.05) in all the corresponding parameters. Soil with oil stress exhibited higher nitrogen (1.5 times) and potassium (1–3 times) content as compared to that of control soil sample. This difference could be attributed to the characteristic feature of soil ecosystems with inherent bioremediation potential [43]. Total organic carbon was found to be 1.05 % and 0.71 % for polluted and pristine soil, respectively.

**Table 1:** Characteristics features of sample for both polluted and control soil

Sr. No	Parameters tested	Polluted soil	Control soil
1	Texture of soil	Fine loamy soil	Fine loamy soil
2	Temperature (°C)	37	37
2	pH	8.10	8.26
3	Organic carbon (%)	1.05	0.71
4	Total Nitrogen (Kgha^−1^)	2070	1371
5	Available P_2_O_5_ (Kgha^−1^)	37.75	26.78
6	Available K_2_O (Kgha^−1^)	170.45	122.21
7	EC (dSm^−1^)	0.33	0.19
8	DNA concentration (ng/μL)	404	358

### Metagenomic DNA extraction and analysis

Soil collected from the industrial area was used for metagenomic DNA extraction using the protocol of Zhou et al. [24]. The obtained DNA was of high molecular weight (Additional file 1: Figure S1) and was pure enough for further sequencing studies. Details about purity ratio and quality of metagenomic DNA (after pooling up DNA for each respective site i.e. P and C) with respect to humic acids is described in Table 1.

Sequencing of two DNA libraries (viz. polluted soil and control soil) was performed and data from the experiments are summarized in Table 2. The sequencing run resulted in 17, 06,040 reads (an average read length of 339 bp) for polluted sample and 39,98,015 reads (an average read length of 356 bp) for control sample. In total 31,284,971 numbers of bases were assembled into 201,285 contigs for polluted soil and 58,039,898 number of bases were assembled into 262,608 contigs for control soil sample using MetaVelvet assembler [26, 27].

**Table 2:** Summary of sequencing result

	Polluted	Control
Number of reads	17,06,040	39,98,015
Average read length	339	356
Total number of contigs	201,285	262,608
Max contig length	1347	1258
Number of bases in contigs	31,284,971	58,039,898
N50	157	221

The sequences were assembled separately using MetaVelvet assembler

### Bacterial diversity analysis

The taxonomic positions of sequenced reads was analysed and studied using two complementary approaches: (1) LCA: classification based on lowest common ancestor using MEGAN [28] and (2) RDP classifier: classification based on 16S rRNA gene sequences [31]. The predominance of bacterial reads was equally observed in all two approaches used to characterize indigenous bacterial community structure with RDP suggesting 97 % dominance of bacteria and 98 % reads were accounted to bacteria in LCA for perturbed environment.

Since 16S rRNA is widely used for taxonomic and phylogenetic studies due to its highly conserved sequences, its hypervariable region can also be used for accurate taxonomic evaluation [44]. The reads assigned to the superkingdom Bacteria are ~97.6 % for polluted and ~95.8 % in pristine indicating dominance of domain bacteria. The niche generated due to perturbed environment was dominated by phylum *Proteobacteria* (60.9 %), followed by *Bacteroidetes* (5.6 %) responsible for lipid metabolism. Only 30 % of *Proteobacteria* and 7.4 % of *Bacteroidetes* was dominant in control sample. The 3rd most abundant taxon in both samples (i.e. polluted and control) is *Verrucomicrobia* (2.4 % and 4.3 %) which was followed by *Firmicutes* (0.6 % and 4.2 %) and *Actinobacteria* (1.3 % and 3.4 %), respectively (Additional file 1: Figure S2).

**Fig. 1:**
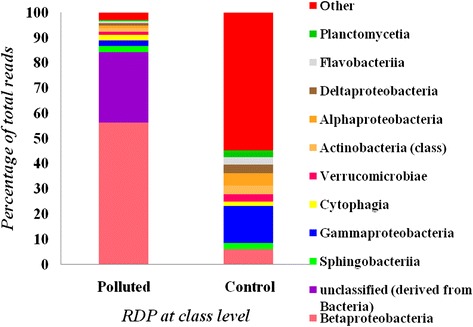
Distribution of taxa among bacteria at rank class classified according to 16S rDNA using RDP classifier for both polluted as well as control sample

At the class level community shift can be clearly seen between polluted and control soil samples. *Gammaproteobacteria* (14.9 %) showed dominancy in control soil while *Betaproteobacteria* (56.3 %) is in abundance for polluted soil sample (Fig. 1). The second most abundant class found in polluted soil is *Sphingobacteria* (2.5 %) followed by *Gammaproteobacteria* (2.2 %), *Cytophagia* (2 %) and *Verrucomicrobiae* (1.4 %). *Betaproteobacteria* (6 %) was found to be second dominant class in control soil sample followed by *Alphaproteobacteria* (5 %), *Actinobacteria* (3.5 %), *Deltaproteobacteria* (3.3 %), *Flavobacteria* (2.9 %) and *Verrucomicrobiae* (2.7 %). At rank genus *Chromobacterium* (45.5 %) (family *Neisseriaceae*) showed dominancy for polluted sample followed by *Neisseria* (4.9 %) (family *Neisseriales)*, *Cupriavidus* (4.5 %) (family *Burkholderiaceae*) and *Pedosphaera* (1.06 %) belonging to the family *Verrucomicrobia.* Genus *Klebsiella* (5 %) (family *Enterobacteriaceae*) was found to be abundant in control sample followed by 2.2 % *Flavobacterium* (family *Flavobacteriaceae*), 1.7 % *Brevibacillus* (family *Paenibacillaceae*) and 1.6 % *Xantomonas* (family *Xanthomanadaceae*). (Data for genus level is not shown)

**Fig. 2:**
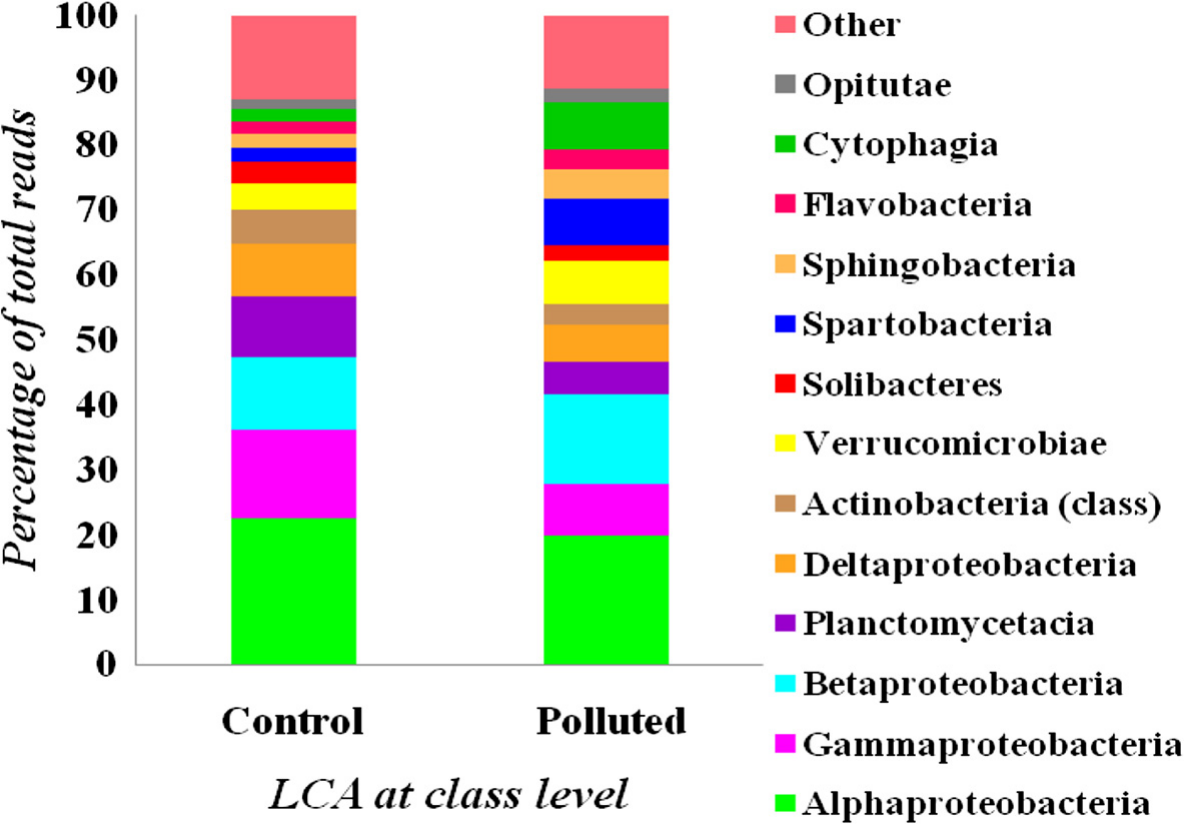
Distribution of taxa among bacteria at rank class classified according to lowest common ancestor (LCA) for both polluted as well as control sample

Meanwhile, we also analyzed microbial community compositions based on lowest common ancestor (LCA). The statistics for both the sample (i.e. polluted and control) are shown in Additional file 1: Figure S3 at rank phylum. However, data for only polluted sample is explained in text. Comparable to the taxonomic structure generated from the output on reads, our analysis revealed that polluted soil sample showed *Proteobacteria* (47 %), followed by *Bacteroidetes* (20 %), *Verrucomicrobia* (15.5 %), *Plantomycetes* (4 %) and *Acidobacteria* (3 %) as most dominant classified phylum (Additional file 1: Figure S3). For polluted site the dominant classes (Fig. 2) in bacteria are *Alphaproteobacteria* (19 %), *Betaproteobacteria* (13 %), *Gammaproteobacteria* (7 %), *Spartobacteria* (7 %) and *Cytophagia* (7 %). At rank genus *Chthoniobacter* (10 %) (family unclassified from *Spartobacteria)*, *Chromobacterium* (7 %) (family *Neisseriaceae*) showed dominancy for polluted sample followed by *Candidatus Solibacter* (3.5 %) (family *Solibacteraceae*), *Verrucomicrobium* (2.9 %) (family *Verrucomicrobiaceae*) and *Chitinophaga* (2.8 %) belonging to the family *Sphingobacteriales.*

In addition to this, the diversity picture for both samples was also compared with EGTs using WebCARMA algorithm and non-redundant database M5NR. The comparison is not described in text but displayed through figures in Additional file 1: Figure S4. Among all the databases used for analysis we found that there is influence of oil contamination on soil and can be clearly seen by the dominance of betaproteobacteria at class level and *Chromobacterium* (widely known for its oil degrading capability) at genus level.

### Comparison of microbial composition between soil samples

**Fig. 3:**
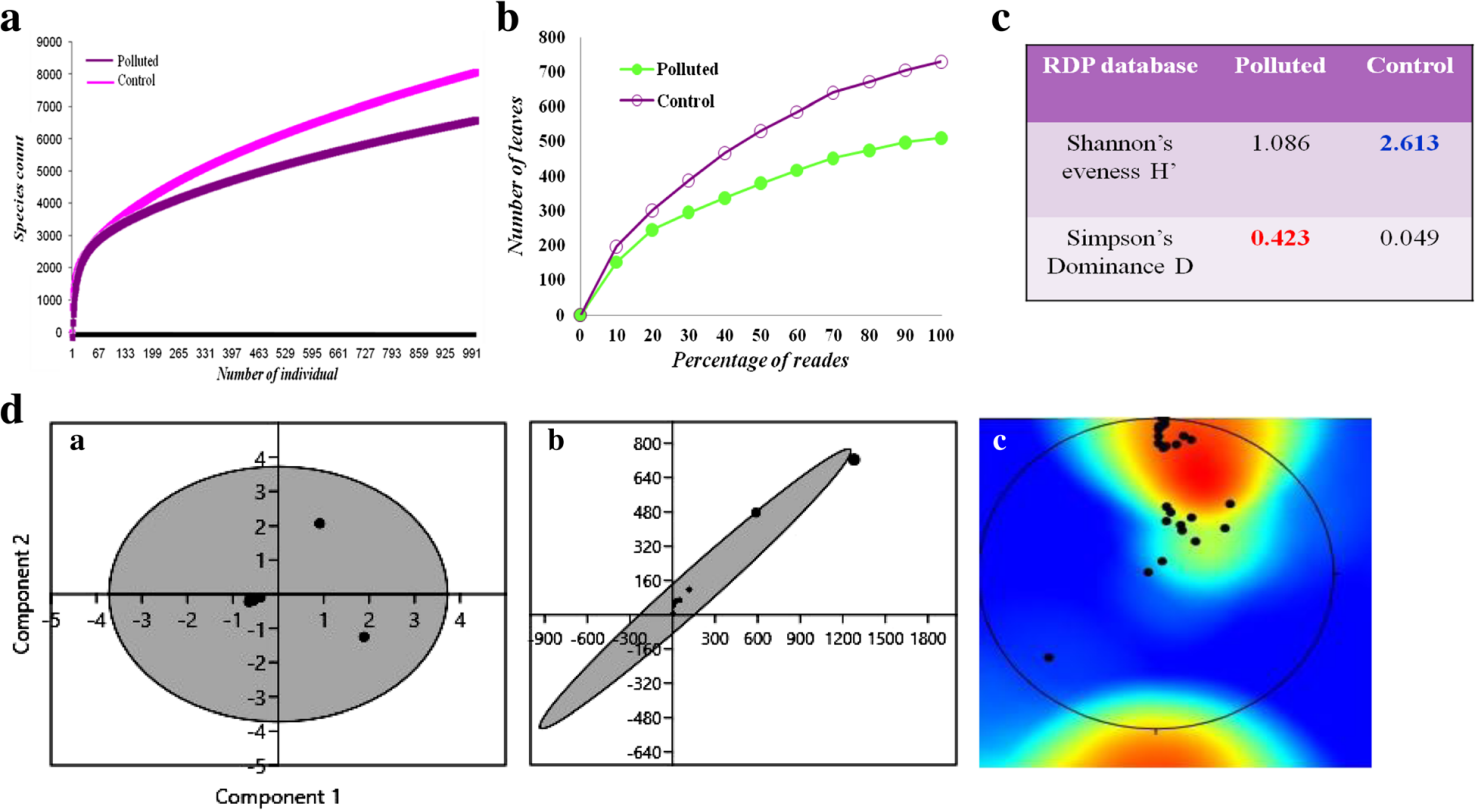
Statistical comparative analysis for the reads assigned between control as well as polluted soil sample (**a**) rarefaction curves on species counts using RDP dataset (**b**) rarefaction curve using percentage of reads, (**c**) diversity indices and (**d**) multivariate analysis *a*) PCA plot, *b*) CA plot, *c*) contour plot

Statistical analysis of biodiversity provides interesting insights as reflected in rarefaction curves. Rarefaction analysis was carried out in order to assess species richness of the system. Using RDP, we analyzed the microbial richness, based on sequence reads, between libraries of polluted and control soil samples (Fig. 3a). Whereas, plotting the number of leaves predicted by LCA algorithm revealed that the number of taxonomic leaves or clades of control soil are all higher than those of polluted ones. Also, control and polluted soil contains 629 and 396 leaves for all assigned taxa, respectively (Fig. 3b). Furthermore, the rarefaction curves of both libraries appear close to saturation at 100 % of the total reads. Our results suggest that the current sampling depth is not yet close to the natural status for bacteria.

Shannon index was used to indicate diversity and complexity, and the Simpson index was used to measure abundance. As exhibited in Fig. 3c, the lowest Shannon diversity in polluted sample indicates presence of phylotypes while in control sample the diversity indices showed a higher level of species richness. Simpson index showed the dominance in polluted sample as compared to that of control. Consistently, the data collected from phylotype distributions of 16S rRNA gene sequences of total bacterial community of both the samples were treated by PCA plot, contour plot and correspondence analysis (CA) plot in order to check differences between the sites in terms of bacterial community structures. The entire three analysis viz. PCA plot, Contour plot and CA plot (Fig. 3d (a, b, c)) was able to separate control site from that of polluted ones. The data set of both the samples showed that sites were well separated from each other and as well as no cluster formation showed difference in bacterial community structure.

### Annotation and mapping of metagenome single reads to the microbial genomes

**Fig. 4:**
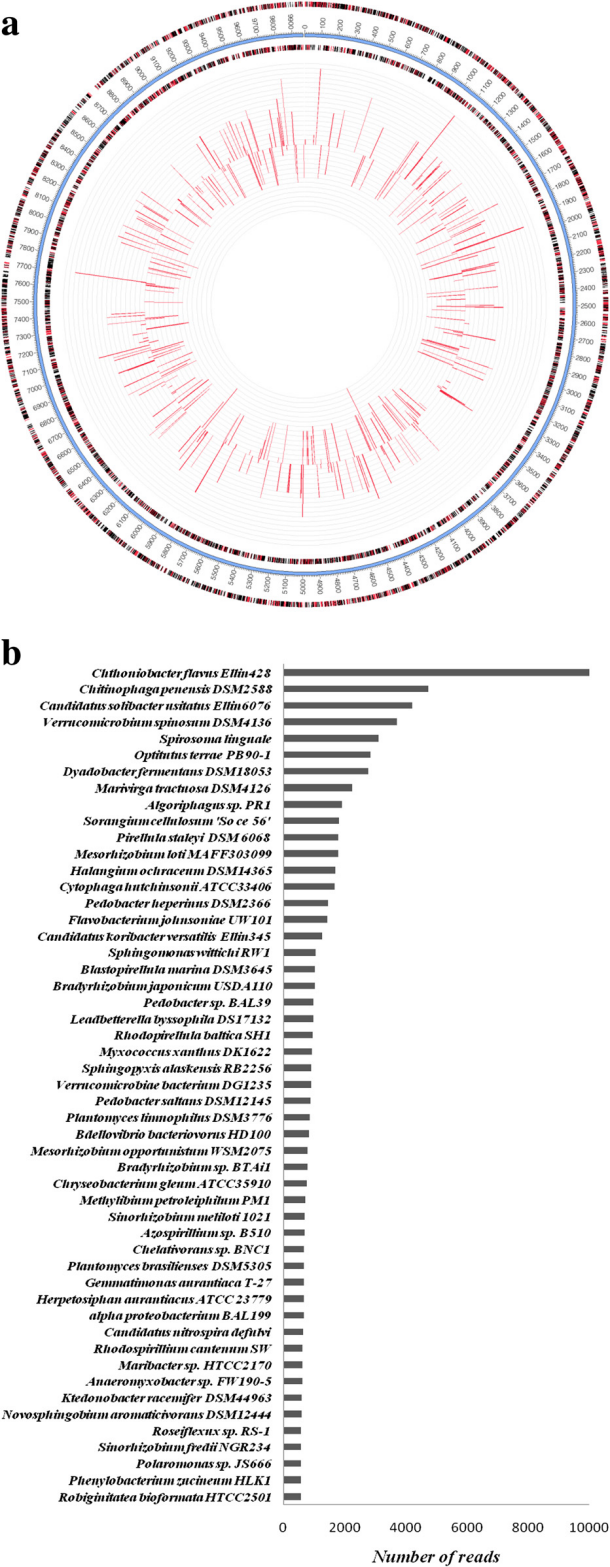
**a** Mapping of metagenomic reads from polluted soil sample on the genome of *Candidatus solibacter ellin* 6076 and **b** Metagenomic reads assigned to microbial genomes sequences. The x-axis denoted the number of reads assigned to the 50 most prevalent microbial strain genomes

Metagenomic reads of polluted sample were mapped for assessing genome coverage. Maximum hits for metagenome were attributed to the genome of *Candidatus solibacter ellin* (Fig. 4a). Maximum identity percentile of metagenomic reads with whole genome of *Candidatus solibacter ellin* was found to be 5.76 %. List of top 50 microorganisms mapped with highest number of reads are shown in Fig. 4b. The highest number of reads was allocated to *Chthoniobacter flavus* Ellin428 genome followed by *Chitinophaga penensis* DSM2588, *Candidatus solibacter* usitatus Ellin6076, *Verrucomicrobium spinosum* DSM4136, *Spirosoma lingual, Optitutus terrae PB90-1, Dyadobacter fermentans DSM18053, Marivirga tractuosa DSM4126.* The list also includes *Algoriphagus, Sorangium, Pirellula, Mesorhizobium, Halangium, Cytophaga* and others. This suggested that these organisms were enriched at polluted site and can play significant role in fatty acid metabolism and synthesis.

### Gene function annotation and classification

**Fig. 5:**
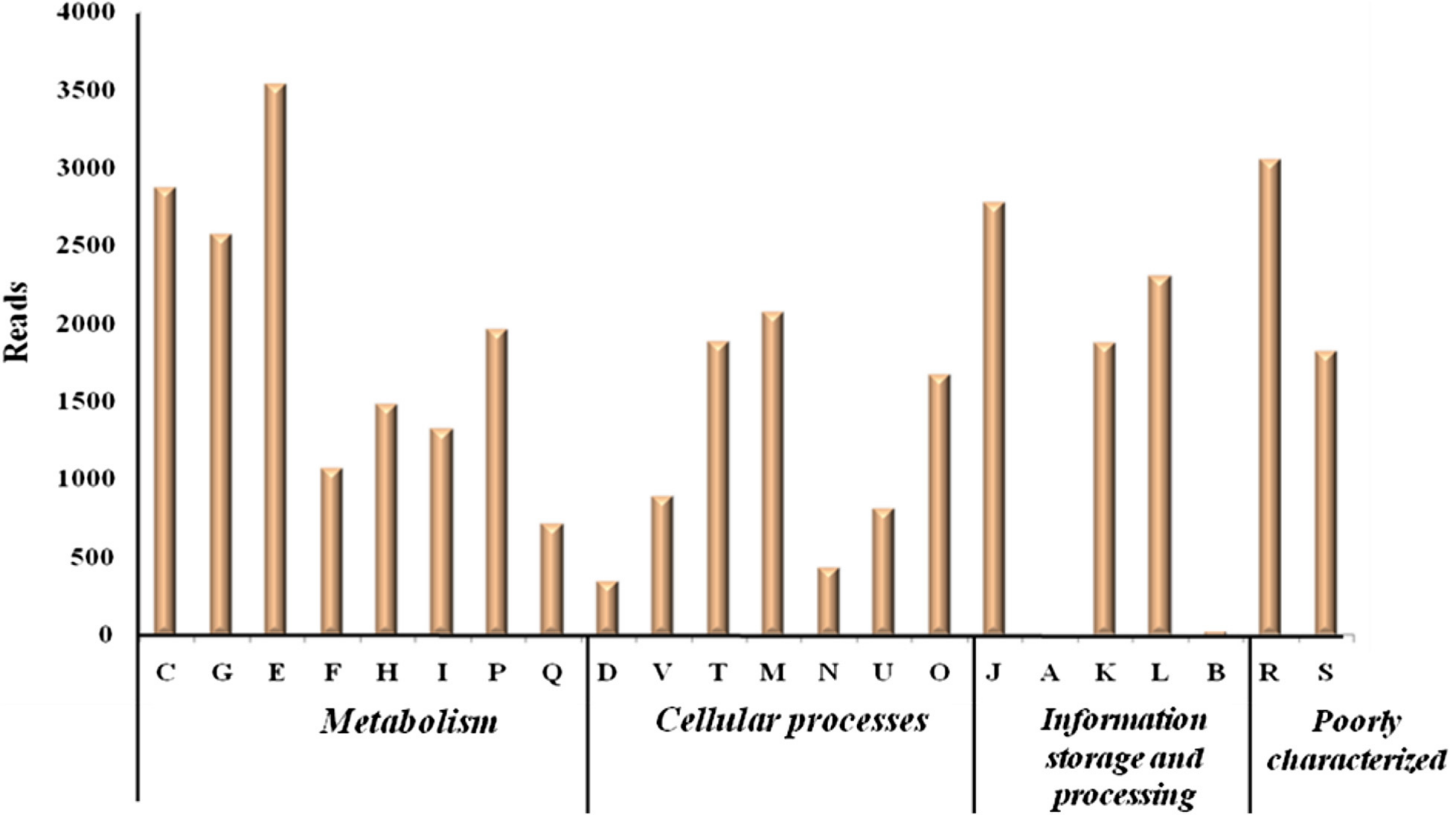
Characterization of metagenomic sequencing reads of contaminated soil sample according to the Cluster of Orthologous Groups of protein (COGs). The categories for COG are abbreviated as follows: C- energy production and conversation; G- carbohydrate transport and metabolism; E- amino acid transport and metabolism; F- nucleotide transport and metabolism; H- coenzyme transport and metabolism; I- lipid transport and metabolism; P- inorganic ion transport and metabolism; Q- secondary metabolites and biosynthesis; D- cell cycle control, cell division, chromosome partitioning; T- signal transduction mechanism; M- cell wall/membrane/envelope biogenesis; N- cell motility; U- intracellular trafficking, secretion and vesicular transport; O- post translational modification, protein turnover, chaperones; J- translation, ribosomal structure and biogenesis; A- RNA processing and modification; K- transcription; L- replication, recombination and repair; B- chromatin structure and dynamics; R- general function prediction only, S- function unknown

**Fig. 6:**
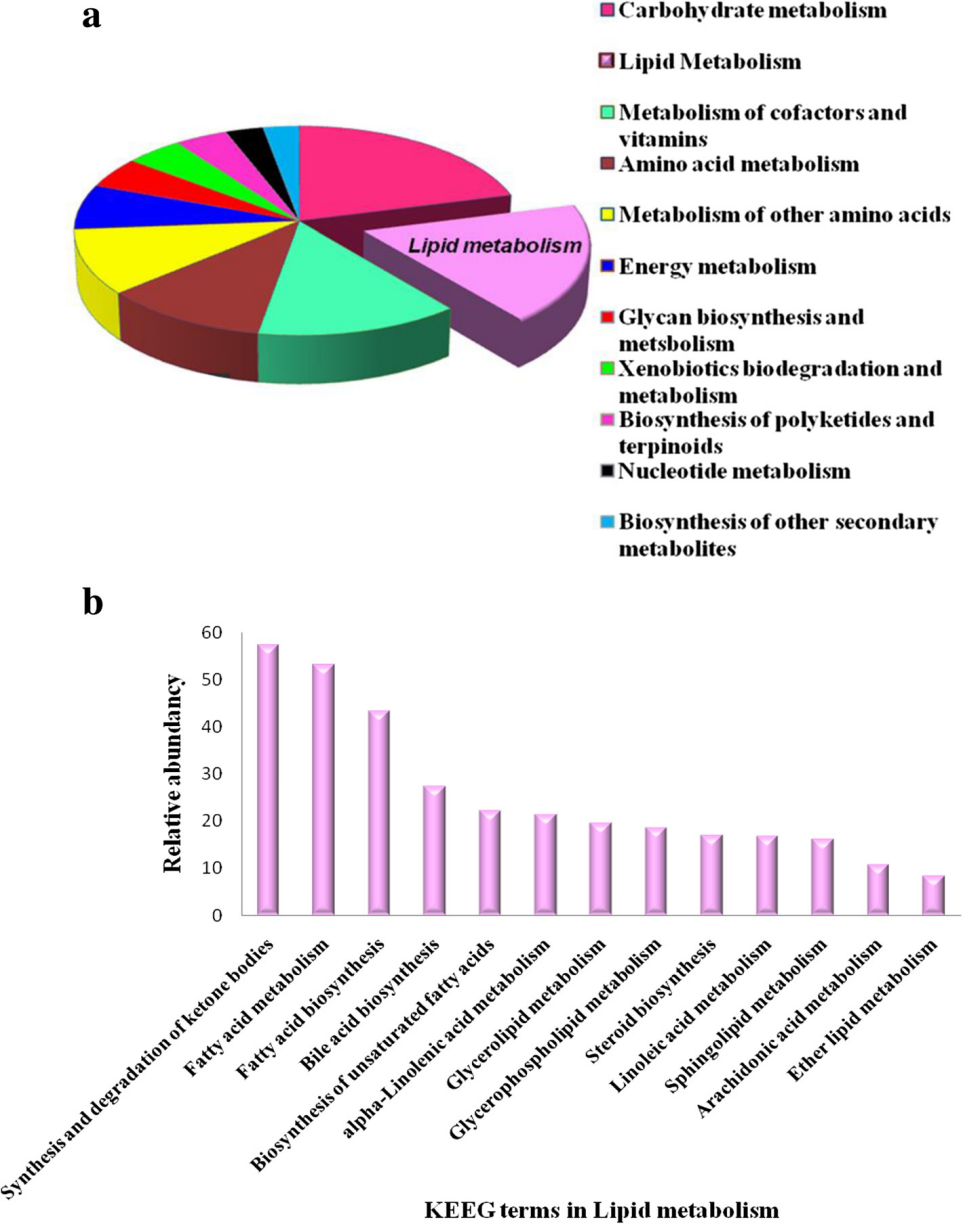
**a** Characterization of metagenomic sequencing reads of contaminated soil sample according to KEEG database. **b** Popular terms on lipid metabolism based on KEEG analysis. The Y-axis refers to the percentage of reads within the reads mapping to metabolism terms

Metabolic profile for bacterial community structure of polluted soil sample was annotated using COG and KEGG databases. Assembled contigs were analyzed by assigning predicted functions to genes based on COG [45]. In total 22 classes based on functional categories were identified by COG database (Fig. 5). In the category “metabolism” large amount of reads are distributed among “amino acid transport and metabolism (E)”, “energy production and conversion (C)”, “carbohydrate transport and metabolism (G)”, and “lipid transport and metabolism (I)”. The class “lipid transport and metabolism (I)” was further characterized for various kinds of enzymes responsible for fatty acid metabolism under stress conditions. Classes such as “inorganic ion transport and metabolism (P)” and “coenzyme metabolism (H),” “secondary metabolites biosynthesis, transport, and catabolism (Q),” and “signal transduction mechanisms (T)” are associated with transport of ions/compounds and other metabolic processes (Fig. 5). COG categories/accessions important in lipid metabolism are described in Table 3. In the KEEG analysis, metabolism term including carbohydrate metabolism, lipid metabolism, metabolism of cofactors and vitamins, amino acid metabolism and metabolism of other amino acids are among the top five most popular categories (Fig. 6a). KEGG terms in lipid metabolism are displayed in Fig. 6b. The enzymes involved in lipid metabolism were detected in reads assigned to fatty acid biosynthesis, glycerophospholipid metabolism, sphingolipid metabolism, glycerolipid metabolism as the four most dominant groups which are involved in the processing of lipids and fatty acids. The 20 most abundant enzymes mapped according to KEGG database from metagenomic data is tabulated in Table 4. This observation is consistent with the findings that many species in polluted sample are involved in fatty acid biosynthesis and fatty acid metabolism.

**Table 3:** COG categories as discovered from the metagenomic reads for lipid metabolism

COG No.	Name of the protein
COG3425	3-hydroxy-3-methylglutaryl CoA synthase
COG0304	3-oxoacyl-(acyl-carrier-protein) synthase
COG0332	3-oxoacyl-[acyl-carrier-protein] synthase III
COG4247	3-phytase (myo-inositol-hexaphosphate 3-phosphohydrolase)
COG1211	4-diphosphocytidyl-2-methyl-D-erithritol synthase
COG0439	Biotin carboxylase
COG2272	Carboxylesterase type B
COG4589	CDP-diglyceride synthetase
COG1024	Enoyl-CoA hydratase/carnithine racemase
COG0821	Enzyme involved in the deoxyxylulose pathway of isoprenoid biosynthesis
COG0657	Esterase/lipase
COG1398	Fatty-acid desaturase
COG1022	Long-chain acyl-CoA synthetases (AMP-forming)
COG3127	Lysophospholipase
COG1443	Isopentenyldiphosphate isomerase
COG2185	Methylmalonyl-CoA mutase, C-terminal domain/subunit (cobalamin-binding)
COG1260	Myo-inositol-1-phosphate synthase
COG2867	Oligoketide cyclase/lipid transport protein
COG0558	Phosphatidylglycerophosphate synthase
COG1562	Phytoene/squalene synthetase
COG3243	Poly(3-hydroxyalkanoate) synthetase
COG4553	Poly-beta-hydroxyalkanoate depolymerase
COG1657	Squalene cyclase
COG0020	Undecaprenyl pyrophosphate synthase

**Table 4:** Abundance of enzymes mapped from metagenomic reads identified in KEEG database

Sr. No	Enzyme mapped	Abundance/Hits
1	ABC transporter related	237
2	binding-protein-dependent transport systems inner membrane component	192
3	TonB-dependent receptor	186
4	TonB-dependent receptor plug	141
5	short-chain dehydrogenase/reductase SDR	119
6	acriflavin resistance protein	118
7	ABC transporter related protein	116
8	NAD-dependent epimerase/dehydratase	114
9	oxidoreductase domain protein	104
10	sulfatase	94
11	protein of unknown function DUF214	90
12	transcriptional regulator	80
13	histidine kinase	76
14	ABC transporter ATP-binding protein	69
15	extracellular solute-binding protein	69
16	inner-membrane translocator	69
17	aldo/keto reductase	68
18	AMP-dependent synthetase and ligase	64
19	beta-lactamase	64
20	glycoside hydrolase family protein	64

The main focus of the study was on fatty acid biosynthesis. The expression of genes involved in lipid metabolism pathways such as fatty acid biosynthesis (PATH: ko00061), fatty acid degradation (PATH: ko00071) mapped from KEEG database are displayed in Additional file 1: Table S1.

### Bacterial community structure using culture dependent approach

Since the contaminated site of the study is oil perturbed soil attempts were made to isolate organisms on tributyrin agar plates containing tributyrin oil as carbon source. Total bacterial counts of 1.8X10^2^ CFU/mL were obtained from oil contaminated soil whereas <30 bacterial counts were obtained from control sample when screened on tributyrin agar plates. The possible reason for this could be production of extracellular enzymes by particular microorganisms to combat these stressed condition in such contaminated environment. Different species of *Bacillus such as (B. subtilis, B. methylotrphicus, B. pumilis, B. endophyticus)*, *Pseudomonas (P. stutzeri, P. sp)* as well as *Exiqobacterium (E. profundum)* known for their lipolytic activities were identified.

## Discussion

### Deciphering bacterial community structure using shotgun sequencing approach

Novel technologies continue to expand our understanding of microbial diversity and community structure. Metagenomic analysis [10, 45] has previously identified ‘unexpectedly’ high bacterial phylogenetic and functional diversity. The long-term sustainability of soil contamination requires detailed knowledge of its biodiversity coupled to profound understanding for its functioning. Previous studies with 16S rRNA-based analyses using clone libraries [46–48], microarrays (for example, PhyloChip and GeoChip) [49–52], pyrosequencing [7, 53] and other approaches [54] showed that soil microbial communities are highly diverse and complex.

Here, we took opportunity to explore microbial diversity and its functioning in edible oil contaminated soil using 16S rRNA shotgun sequencing approach. This study provides a comprehensive survey of the microbial richness and composition of long-term oil contaminated soil microbial communities. Upon taxonomic analysis using different approaches (RDP classifier and LCA algorithm), *Proteobacteria* was the well-represented phylum along with β-, α-, γ-, and δ-*Proteobacteria*. This group of bacteria has considerable morphological, physiological and metabolic diversity, which are of great importance to global carbon, nitrogen and sulfur cycling [50]. *Bacteroidetes* are the second most prevalent group of bacteria detected in polluted sample, with three major classes (*Sphingobacteria, Cytophagia and Flavobacteriia*). *Gammaproteobacteria* is dominant group of bacteria followed by β-, α-*Proteobacteria* in pristine soil sample. The results showed significantly altered microbial community diversity, composition and structure, especially for particular microbial populations at class level.

The members of the *Proteobacteria* phylum are a group of Gram-negative bacteria that have an important role in decomposition of organic matter and carbon cycling [53]; *Neisseriaceae* and *Burkholderiaceae* were found to be major families. *Proteobacteria* has been previously detected at high abundance in soil samples, including polluted ones [55–58], and shift in their community were also observed upon contamination with oil or during bioremediation. While, *Proteobacteria* accounted for 60 % of total sequences in our polluted soil sample, they accounted for 86 % in long-term diesel-contaminated soil from Poland [58], 45 % in contaminated permafrost soils along a crude oil pipeline in china [59], 42 % in gradient of petroleum contaminated desert soil [17] and 50-60 % in contaminated mangrove sediments from Brazil [57]. Members of *Betaproteobacteria* and *Gammaproteobacteria* are known to be highly versatile for their degradation ability [7, 60, 61]. The strains of genera *Chromobacterium, Xanthomonas, Pseudomonas, Burkholderia* and *Acenitobacter* which prevailed the classes *Betaproteobacteria* and *Gammaproteobacteria* were found to possess oil-degrading capabilities [17, 18, 22]. Various microbial populations that are capable of degrading different oil and petroleum products including species of *Pseudomonas, Flavobacterium, Arthrobacter, Alcaligenes, Nocardia, Micrococcus, Corynebacterium* and *Mycobacterium* have been isolated from soil [62], while *Pseudomonas, Arthrobacter, Sphingomonas, Rhodococcus, Ochrobactrum, Psychrobacter, Pseudoalteromonas, Acinetobacter* and *Bacillus* are isolated from marine environment [63–66]. Among the detected genera known for degradation are *Actinobacteria*, *Microbacterium* and *Micrococcus* [40, 67].

Rarefaction analysis for particular ecosystem is a prerequisite to deduce the complete taxonomic profile of the community. The rarefaction curves are nearly reaching saturation for classifications based on RDP. Moreover, results from PCA plot, CA plot and contour plot also suggested there is variation in community structure for both samples and appropriate depth of sampling is also covered. Mapping of metagenome reads onto bacterial genomes suggested that organisms related to the identified species were enriched at the site contaminated with oil and presumably play an active role in biodegradation.

Understanding the factors that influence microbial community structure is an important goal in microbial ecology [51]. Our analysis results indicate that oil has a significant impact on soil microbial functional communities in contaminated soil. On one hand oil contamination could be toxic to many microbial populations reducing microbial diversity and on the other hand, the vast range on carbon substrates and subsequent metabolites present in oil-contaminated soil could facilitate the development of rather complex microbial communities. In this study, microbial functional genes encoded for lipid metabolism was analyzed using KEEG database [39]. The pathways of fatty acid biosynthesis and the enzymes involved in them are well conserved. The abundance of several functional genes involved in fatty acid synthesis and metabolism such as *acc*, *fab* and *fad* genes were detected. These genes are directly involved in the synthesis and metabolism of free fatty acids [68]. The increase in these functional genes might be due to the natural selection of organisms capable of utilizing fatty acids/lipids. The degradation of PAHs by microorganisms through a complex enzymatic process was well documented by Patel et al. [63] with increase in frequencies of *nahA* genes in polluted water. Bestawy et al. [22] reported a positive correlation between oil contamination and abundance of *fab* genes from gram-negative bacteria removing oil and grease in industrial effluent.

### Metabolic pathway analysis for fatty acid biosynthesis

**Fig. 7:**
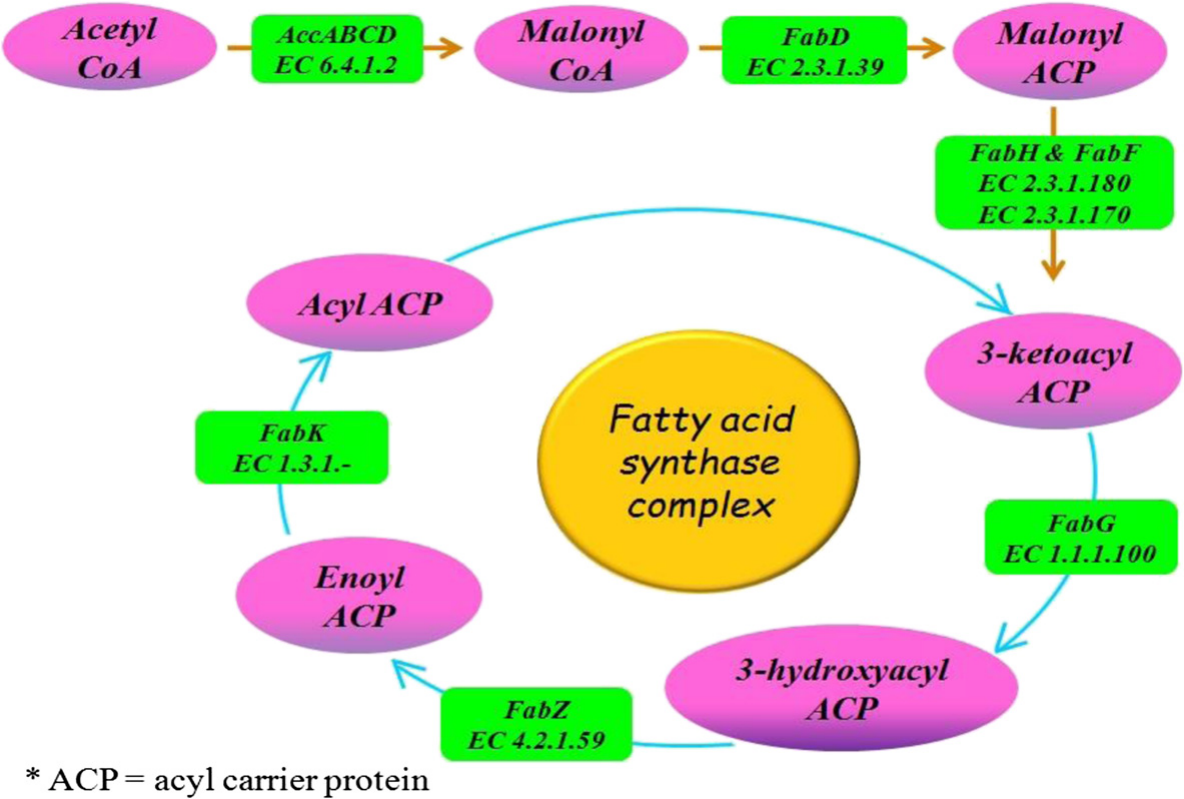
Fatty acid synthesis and metabolism pathway predicted in polluted soil sample based on KEEG analysis. Pink colors indicate formation of compounds within the reactions and green labels indicates presence of genes detected in metagenomic reads

Fatty acid biosynthesis in almost all the organisms culminates in formation of saturated fatty acids. All organisms produce fatty acids via a repeated cycle of reactions involving the condensation, reduction, dehydration and reduction of carbon-carbon bonds. First step in fatty acid biosynthesis (Fig. 7) is the ATP dependent formation of malonyl-CoA from acetyl-CoA and bicarbonate by acetyl-CoA carboxylase (*acc*, EC 6.4.1.2) enzyme. All bacterial organisms contain a type II synthase (FAS II) for which each reaction is catalyzed by a discrete protein and reaction intermediates are carried in the cytosol as thioesters of the small acyl carrier protein (ACP) [45]. Malonyl CoA:ACP transferase encoded by *fabD* gene (EC 2.3.1.39) undergoes transacylation of malonyl from CoA to ACP. The chain elongation step in fatty acid biosynthesis consists of the condensation of acyl groups, which are derived from acyl- CoA or acyl-ACP, with malonyl-ACP by two types of 3-ketoacyl-ACP synthases. The first class of 3-ketoacyl-ACP synthase III (*fabH*, EC 2.3.1.180) is responsible for the initiation of fatty acid elongation and utilizes acyl-CoA primers. The second class of enzymes (*fabF* EC 2.3.1.179 and *fabB,* EC 2.3.1.41) is responsible for the subsequent rounds of fatty acid [68–70]. Thus, produced acyl-ACP is catalyzed by three enzymes [NADPH-dependent 3-ketoacyl-ACP reductase (f*abG*, EC 1.1.1.100), 3-hydroxyacyl-ACP dehydratase (*fabZ,* EC 4.2.1.59) and NAD(P)H-dependent enoyl-ACP reductase (*fabI,* EC 1.3.1.9, 1.3.1.10)] for reduction, dehydration and reduction of carbon-carbon bonds,respectively. Further, additional cycles are initiated by *fabF* and *fabB*. The *fad*R_Ec_ protein is a global regulator of fatty acid degradation, is a transcriptional activator that binds to the *fabA* promoter region [71].

Finally, input to fatty acid synthesis is acetyl CoA and the output is free fatty acid synthesis. The *fab* and *fad* proteins are highly conserved in many grampositive bacteria including *Bacillus*, *Clostridium*, *Streptomyces* and other related genera [70–72]. Moreover, its orthologues are unexpectedly present in more diverse genera, such as *Metanosarcina* (*Archaea*), and *Bordetella*, *Burkholderia* and *Chromobacteria* (*β*-proteobacteria) [70]. Also, genome analysis indicated that only the α-, β and γ-proteobacteria have the proteins of this pathway [73, 74].

### Bacterial isolates by culture dependent approach

Using culturable approach higher number of total bacterial count was observed in presence of tributyrin from polluted environment in comparison to that of pristine. Result clearly indicates the adaption of organism toward oil stress. Biodegradation by intrinsic microbial populations is the key and reliable system through which thousands of organic contaminants are eradicated from the environment [75]. Different species of *Bacillus* (*B. subtilis, B. pumilis, B. endophyticus)* and *Pseudomonas (P. stutzeri, Pseudomonas sps.)* were found in abundance from oil stressed soil which depicts their significant role in degradation of oil. These microbial strains have ability for producing extracellular lipase enzymes that hydrolyze triglycerides (the main component of oils and fats) to fatty acids and glycerol [21, 22]. The enzymatic versatility of these bacteria is well known and has been suggested as their importance in ecosystem. As noted by Ahmad et al., *Bacillus* strains play an important role in biodegradation of oil contaminated soil in combination with that of *Pseudomonas sp* [41]. Apart from this species such as B*. thuringiensis, Micrococcus sp, Corynebacterium sp.,* and *Acinetobacter sp.* also has significant role in degradation of pollutants [75, 76].

## Conclusion

In conclusion, present study reflects the detection of microbial diversity across oil stress condition favouring *β-proteobacteria* such as *Chromobacterium, Xanthomonas, Pseudomonas, Burkholderia* and *Acinetobacter sp.* The microbial community analysis at the metagenome level gives an insight into the repertoire of species to deal with oil contamination. We also observed, genes corresponding to enzymes involved in a wide variety of reactions and operating in many unrelated biosynthesis pathways collaborates well with the fact that the site of study has long-term oil contamination. Moreover, the isolation of different genera of *β-proteobacteria* is in correspondence with results obtained by culture dependent approach reporting abundance of *β-proteobacteria* in polluted samples. These isolates are well documented for biodegradation processes. In this regard, obtained knowledge will be useful in understanding the pathways for synthesis and metabolism of fatty acids released for oils and the microbial communities dominating in such stress condition.

### Availability of supporting data

The sequence data for both soil samples i.e. polluted and control obtained from Ion Torrent PGM platform has been deposited at MGRAST server (version 3). MGRAST IDs for the datasets are 4508969.3 and 4516462.3 for polluted soil and control soil, respectively. MGRAST IDs for the contig obtained from both the samples are 4515485.3 and 4512472.3, respectively. The DOI link for the server is http://metagenomics.anl.gov/metagenomics.cgi?page=Home. The sequences obtained from the culturable diversity study have been submitted to GenBank, NCBI and their accession numbers are from KR140170 to KR140186 (polluted soil) and KR140187 to KR140201 (control soil). The DOI link for GenBank is http://www.ncbi.nlm.nih.gov/genbank.

## Additional file

Additional file 1: Figure S1. Metagenomic DNA extracted from polluted as well as control soil sample and electrophoresed on 0.8 % agarosa gel. Lane M is of marker, Lane 1, is for polluted sample (representing pooled metagenomic DNA for P1 + P2 + P3 = P) and Lane 2 is for control soil sample (representing pooled metagenomic DNA for C1 + C2 + C3 = C). **Figure S2.** Distribution of taxa among bacteria at rank phylum classified according to 16S rDNA using RDP classifier for both polluted as well as control sample. **Figure S3.** Distribution of taxa among bacteria at rank phylum classified according to lowest common ancestor (LCA) for both polluted as well as control sample. **Figure S4.** Comparative distribution of taxa among bacteria at rank class classified according to WebCARMA and M5NR datasets for both polluted as well as control sample. **Table S1.** Enzymes mapped for lipid metabolism pathways in KEEG database. (DOC 300 kb)
