# Supplementary material for: Response and resilience of soil microbial communities inhabiting in edible oil stress/contamination from industrial estates
Source: BMC Microbiol. 2016 Mar 22;16:50. doi: 10.1186/s12866-016-0669-8 (PMC4802719; doi:10.1186/s12866-016-0669-8)
Supplement: Additional file 1: Figure S1. — Metagenomic DNA extracted from polluted as well as control soil sample and electrophoresed on 0.8 % agarosa gel. Lane M is of marker, Lane 1, is for polluted sample (representing pooled metagenomic DNA for P1 + P2 + P3 = P) and Lane 2 is for control soil sample (representing pooled metagenomic DNA for C1 + C2 + C3 = C). Figure S2. Distribution of taxa among bacteria at rank phylum classified according to 16S rDNA using RDP classifier for both polluted as well as control sample. Figure S3. Distribution of taxa among bacteria at rank phylum classified according to lowest common ancestor (LCA) for both polluted as well as control sample. Figure S4. Comparative distribution of taxa among bacteria at rank class classified according to WebCARMA and M5NR datasets for both polluted as well as control sample. Table S1. Enzymes mapped for lipid metabolism pathways in KEEG database. (DOC 300 kb) [file 12866_2016_669_MOESM1_ESM.doc]

Supplementary Information

**Response and resilience of soil microbial communities inhabiting in edible oil stress/contamination from industrial estates**

*Vrutika Patel^1^, Anukriti Sharma^2^, Rup Lal^2^, Naif Abdullah Al-Dhabi^3^ and Datta Madamwar^1^*

1. *Post Graduate Department of Biosciences, Centre of Advanced Study in Bioresource Technology, Sardar Patel University, Satellite Campus, Vadtal Road, Bakrol - 388 315, Gujarat, India.*
2. *Department of Zoology, University of Delhi, Delhi, India.*
3. *Department of Botany and Microbiology, Addiriya Chair for Environmental Studies, College of Science, King Saud University, P.O. Box # 2455, Riyadh 11451, Saudi Arabia.*

**** Author for correspondence:***

Prof. Datta Madamwar

Tel: +91 2692 229380

Fax: +91 2692 236475

E-mail address: datta_madamwar@yahoo.com (Datta Madamwar)

[vrutikaptl_19@yahoo.com](mailto:vrutikaptl_19@yahoo.com) (Vrutika Patel)

[anukriti.sharma8@gmail.com](mailto:anukriti.sharma8@gmail.com) (Anukriti Sharma)

[ruplal@gmail.com](mailto:ruplal@gmail.com) (Rup Lal)

[naldhabi@ksu.edu.sa](mailto:naldhabi@ksu.edu.sa) (Naif Abdullah Al-Dhabi)


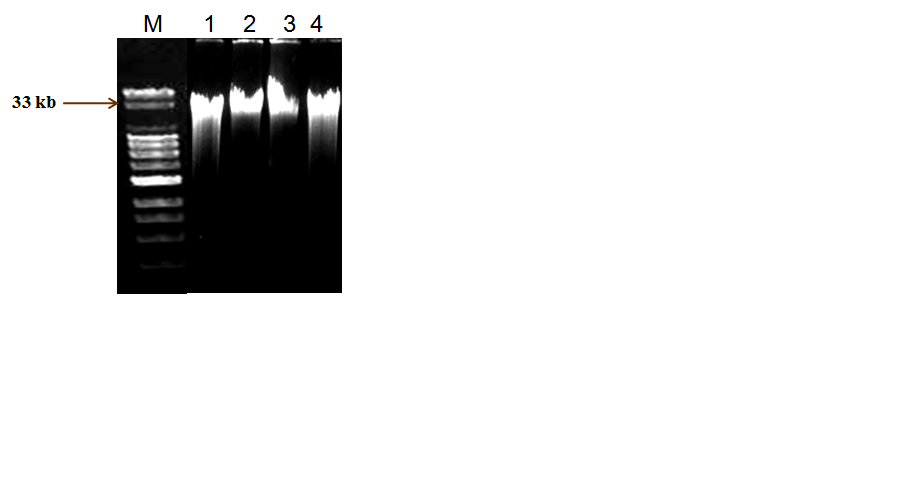


**Figure S1:** Metagenomic DNA extracted from polluted as well as control soil sample and electrophoresed on 0.8% agarosa gel. Lane M is of marker, Lane 1 is for polluted soil sample (representing polled metagenomic DNA for P1+P2+P3=P) and Lane 2 is for control soil sample (representing polled metagenomic DNA for C1+C2+C3=C).


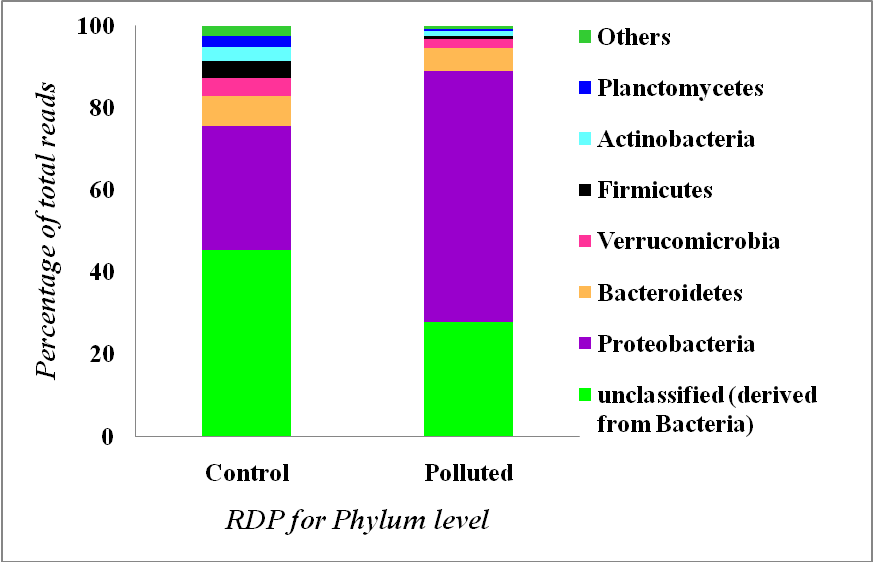


**Figure S2:** Distribution of taxa among bacteria at rank phylum classified according to 16S rDNA using RDP classifier for both polluted as well as control sample.


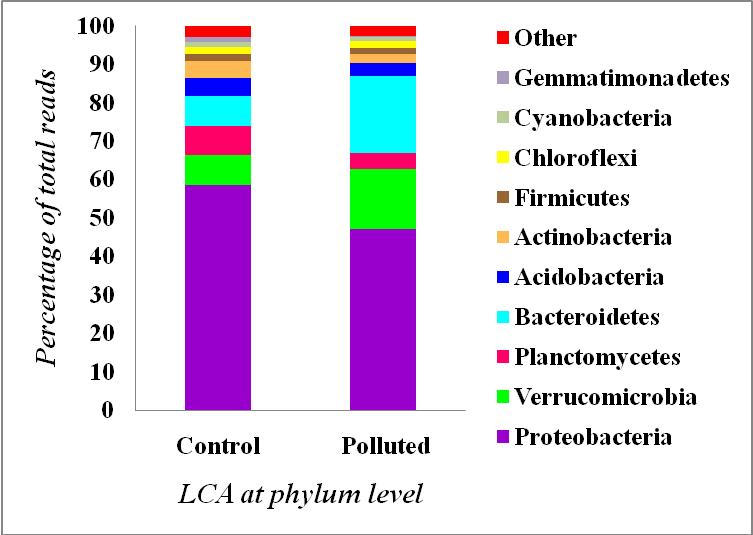


**Figure S3:** Distribution of taxa among bacteria at phylum classified according to lowest common ancestor (LCA) for both polluted as well as control sample.

**Figure S4:** Comparative distribution of taxa among bacteria at rank class classified according to WebCARMA and M5NR datasets for both polluted as well as control sample.

**Table S1:** Enzymes mapped for lipid metabolism pathways in KEEG database

| ***Lipid metabolism*** | ***Enzyme*** |
| --- | --- |
| Glycerophospholipid metabolism [PATH:ko00564] | NMT; phosphoethanolamine N-methyltransferase [EC:2.1.1.103] |
| Fatty acid biosynthesis [PATH:ko00061] | DESA1; acyl-[acyl-carrier-protein] desaturase [EC:1.14.19.2] |
|  | DESA2; acyl-[acyl-carrier-protein] desaturase [EC:1.14.19.2] |
|  | oleoyl-[acyl-carrier-protein] hydrolase [EC:3.1.2.14] |
|  | FAS1; fatty acid synthase subunit beta, fungi type [EC:2.3.1.86] |
|  | fabB; 3-oxoacyl-[acyl-carrier-protein] synthase I [EC:2.3.1.41] |
|  | fabD; [acyl-carrier-protein] S-malonyltransferase [EC:2.3.1.39] |
|  | fabF; 3-oxoacyl-[acyl-carrier-protein] synthase II [EC:2.3.1.179] |
|  | fabG; 3-oxoacyl-[acyl-carrier protein] reductase [EC:1.1.1.100] |
|  | fabH; 3-oxoacyl-[acyl-carrier-protein] synthase III [EC:2.3.1.180] |
|  | fabI; enoyl-[acyl-carrier protein] reductase I [EC:1.3.1.9 1.3.1.10] |
|  | fabK; enoyl-[acyl-carrier protein] reductase II [EC:1.3.1.-] |
|  | fabZ; 3-hydroxyacyl-[acyl-carrier-protein] dehydratase [EC:4.2.1.59] |
|  | fas; fatty acid synthase, bacteria type [EC:2.3.1.-] |
| Fatty acid metabolism [PATH:ko00071] | ACADL; long-chain-acyl-CoA dehydrogenase [EC:1.3.8.8] |
|  | DCI; 3,2-trans-enoyl-CoA isomerase, mitochondrial [EC:5.3.3.8] |
|  | alkane 1-monooxygenase [EC:1.14.15.3] |
|  | fadE; acyl-CoA dehydrogenase [EC:1.3.99.-] |
| Steroid biosynthesis [PATH:ko00100] | SC4MOL, ERG25; methylsterol monooxygenase [EC:1.14.13.72] |
|  | TM7SF2, ERG24; delta14-sterol reductase [EC:1.3.1.70] |
|  | 24-methylenesterol C-methyltransferase [EC:2.1.1.143] |
|  | SMT1, ERG6; sterol 24-C-methyltransferase [EC:2.1.1.41] |
| Primary bile acid biosynthesis [PATH:ko00120] | AKR1D1; 3-oxo-5-beta-steroid 4-dehydrogenase [EC:1.3.1.3] |
|  | HSD3B7; cholest-5-ene-3beta,7alpha-diol 3beta-dehydrogenase [EC:1.1.1.181] |
| Steroid hormone biosynthesis [PATH:ko00140] | CYP11B1; cytochrome P450, family 11, subfamily B1 |
|  | CYP21; cytochrome P450, family 21, subfamily A steroid 21-monooxygenase) [EC:1.14.99.10] |
|  | aslA; arylsulfatase [EC:3.1.6.1] |
|  | HSD17B1; 17beta-estradiol 17-dehydrogenase [EC:1.1.1.62] |
| Glycerolipid metabolism [PATH:ko00561] | glpK; glycerol kinase [EC:2.7.1.30] |
|  | mdoB; phosphoglycerol transferase [EC:2.7.8.20] |
|  | GAT; glycerol-3-phosphate O-acyltransferase / dihydroxyacetone phosphate acyltransferase [EC:2.3.1.15 2.3.1.42] |
|  | dhaK; dihydroxyacetone kinase, N-terminal domain [EC:2.7.1.-] |
|  | dhaT; 1,3-propanediol dehydrogenase [EC:1.1.1.202] |
|  | plsX; glycerol-3-phosphate acyltransferase PlsX [EC:2.3.1.15] |
|  | plsY; glycerol-3-phosphate acyltransferase PlsY [EC:2.3.1.15] |
|  | ugtP; 1,2-diacylglycerol 3-glucosyltransferase [EC:2.4.1.157] |
| Glycerophospholipid metabolism [PATH:ko00564] | ACHE; acetylcholinesterase [EC:3.1.1.7] |
|  | CHPT1, CPT1; diacylglycerol cholinephosphotransferase [EC:2.7.8.2] |
|  | CKI1; choline kinase [EC:2.7.1.32] |
|  | alpha-glycerophosphate oxidase [EC:1.1.3.21] |
|  | ETNK, EKI; ethanolamine kinase [EC:2.7.1.82] |
|  | PCYT1; choline-phosphate cytidylyltransferase [EC:2.7.7.15] |
|  | PCYT2; ethanolamine-phosphate cytidylyltransferase [EC:2.7.7.14] |
|  | PLA2G, SPLA2; secretory phospholipase A2 [EC:3.1.1.4] |
|  | TAZ; monolysocardiolipin acyltransferase [EC:2.3.1.-] |
|  | eutB; ethanolamine ammonia-lyase large subunit [EC:4.3.1.7] |
|  | eutC; ethanolamine ammonia-lyase small subunit [EC:4.3.1.7] |
|  | glpA, glpD; glycerol-3-phosphate dehydrogenase [EC:1.1.5.3] |
|  | glpC; glycerol-3-phosphate dehydrogenase subunit C [EC:1.1.5.3] |
|  | gpsA; glycerol-3-phosphate dehydrogenase NAD P)+) [EC:1.1.1.94] |
|  | pldA; phospholipase A1 [EC:3.1.1.32 3.1.1.4] |
|  | pldB; lysophospholipase [EC:3.1.1.5] |
|  | pmtA; phosphatidylethanolamine/phosphatidyl-N-methylethanolamine N-methyltransferase [EC:2.1.1.17 2.1.1.71] |
| Arachidonic acid metabolism [PATH:ko00590] | ALOX15B; arachidonate 15-lipoxygenase second type) / 8-lipoxygenase S-type) [EC:1.13.11.33 1.13.11.-] |
|  | prostaglandin-F synthase [EC:1.1.1.188] |
|  | glutathione peroxidase [EC:1.11.1.9] |
|  | LTA4H; leukotriene-A4 hydrolase [EC:3.3.2.6] |
|  | PTGES2; microsomal prostaglandin-E synthase 2 [EC:5.3.99.3] |
|  | PTGS1, COX1; prostaglandin-endoperoxide synthase 1 [EC:1.14.99.1] |
|  | ggt; gamma-glutamyltranspeptidase [EC:2.3.2.2] |
| Linoleic acid metabolism [PATH:ko00591] | E1.14.19.3; linoleoyl-CoA desaturase [EC:1.14.19.3] |
|  | E1.14.99.33; delta12-fatty acid dehydrogenase [EC:1.14.99.33] |
|  | AOS; hydroperoxide dehydratase [EC:4.2.1.92] |
| Sphingolipid metabolism [PATH:ko00600] | ACER, ASAH3; alkaline ceramidase [EC:3.5.1.23] |
|  | B4GALT6; beta-1,4-galactosyltransferase 6 [EC:2.4.1.274] |
|  | CGT, UGT8; 2-hydroxyacylsphingosine 1-beta-galactosyltransferase [EC:2.4.1.45] |
|  | 3-dehydrosphinganine reductase [EC:1.1.1.102] |
|  | CERK; ceramide kinase [EC:2.7.1.138] |
|  | ENPP7; ectonucleotide pyrophosphatase/phosphodiesterase family member 7 [EC:3.1.4.12] |
|  | LAG1; Acyl-CoA-dependent ceramide synthase [EC:2.3.1.24] |
|  | SGMS; shingomyelin synthase [EC:2.7.8.27] |
|  | SMPD3; sphingomyelin phosphodiesterase 3 [EC:3.1.4.12] |
|  | SMPD4; sphingomyelin phosphodiesterase 4 [EC:3.1.4.12] |
|  | SUR2; C4-hydroxylase [EC:1.14.-.-] |
|  | UGCG; ceramide glucosyltransferase [EC:2.4.1.80] |
| Biosynthesis of unsaturated fatty acids [PATH:ko01040] | E3.1.2.-; [EC:3.1.2.-] |
|  | SCD, desC; stearoyl-CoA desaturase delta-9 desaturase) [EC:1.14.19.1] |
|  | yciA; acyl-CoA thioesterase YciA [EC:3.1.2.-] |
